# Action of ear acupuncture in people with chronic pain in the spinal column: a randomized clinical trial^1^

**DOI:** 10.1590/1518-8345.2678.3050

**Published:** 2018-09-03

**Authors:** Caroline de Castro Moura, Denise Hollanda Iunes, Silvia Graciela Ruginsk, Valéria Helena Salgado Souza, Bianca Bacelar de Assis, Erika de Cássia Lopes Chaves

**Affiliations:** 2 Doctoral student, Universidade Federal de Minas Gerais, Belo Horizonte, MG, Brazil.; 3 PhD, Associate Professor, Departamento de Fisioterapia, Universidade Federal de Alfenas, Alfenas, MG, Brazil.; 4 PhD, Adjunct Professor, Departamento de Ciências Fisiológicas, Universidade Federal de Alfenas, Alfenas, MG, Brazil.; 5 MSc, Professor, Departamento de Enfermagem, Faculdade de Ciências e Tecnologias de Campos Gerais, Campos Gerais, MG, Brazil.; 6 Master’s student, Universidade Federal de Alfenas, Alfenas, MG, Brazil.; 7 PhD, Adjunct Professor, Escola de Enfermagem, Universidade Federal de Alfenas, Alfenas, MG, Brazil.

**Keywords:** Chronic Pain, Back Pain, Acupuncture, Ear, Rehabilitation, Complementary Therapies, Nursing

## Abstract

**Objectives:**

to assess the action of ear acupuncture on disability and tissue temperature in people with chronic pain in the spinal column.

**Method:**

a clinical trial with a sample of 110 people, randomized into three groups: Treatment, Placebo and Control. The assessment instruments were the Rolland Morris Disability Questionnaire (RMDQ) and a thermographic camera, administered before the first treatment session, one week after and 15 days after (follow-up) the fifth session of ear acupuncture. In the analysis of the data, the Kruskal Wallis, Student-Newman Keuls and Wilcoxon tests were applied.

**Results:**

there was a significant reduction in disability in the Treatment and Placebo groups between the initial and final assessments (p<0.05) and between the initial assessments and follow-up (p<0.05). In the final assessment, the Treatment group presented improvement of disability when compared with the Placebo and Control groups (p<0.05). There was an increase in mean tissue temperature of the dorsal region between the initial and follow-up assessments in Treatment and Control groups (p<0.05), and between the final assessments and follow-up in the Treatment and Placebo groups (p<0.05).

**Conclusion:**

ear acupuncture was efficacious in reducing disability and increasing tissue temperature in people with chronic pain in the spinal column. Brazilian Register of Clinical Trials (RBR-5X69X2).

## Introduction

Pain is characterized as the organism’s response to aggression or any pathological disturbance. When it becomes chronic, it is considered a pathology in itself and is prevalent at a high level throughout the world, principally when it affects the lumbar region^1^. 

The severity and chronic nature of back pain are associated with severe functional limitations^2^. In people with disorders of the musculoskeletal system, particularly in the lower limbs and in the lumbar region, impaired physical mobility is one of the most frequent consequences^3^, causing serious compromise in these people’s daily routine, resulting in a high degree of dependency^3^ ^-^ ^4^. 

Assessing chronic pain and its consequences, as well as its treatment, is a major challenge; as this is a subjective phenomena, it is important to invest in studies which can investigate not only the intensity of the pain, but also its implications in people’s lives. 

The Rolland Morris Disability Questionnaire (RMDQ)^5^ is one of the most-used instruments for assessing inability to undertake activities of daily living among people with chronic pain in general^6^. It allows an appropriate assessment of the treatment and of the progression of the patients with chronic pain, through quantifying the limitations caused by this, in both physical and mental functions^5^.

In addition to the behavioral approach, commonly used in investigations of chronic pain, it is necessary to identify the physiological aspects involved in the process of recognizing the painful neuromuscular conditions. In this regard, change in tissue temperature is an important aspect for assessment, as this reflects the kinetic energy of the individual molecules in accordance with simultaneous recruitment of the mechanisms of heat retention and loss^7^. 

The skin is an efficient system for controlling heat. The conducting of heat through this organ is controlled by the degree of vasoconstriction of the arterioles, which supply the cutaneous venous plexus with blood. This vasoconstriction is under control of the sympathetic input of the autonomous nervous system, and takes place in response to changes in the central temperatures of the body and of the environment^8^. Thus, variations in the skin temperature may reflect internal changes, particularly in the activity of the skeletal muscle. 

Another approach indicated for assessing painful conditions, therefore, which extends the resources for measuring the same, is infrared thermography - an imaging test which is of proven safety, noninvasive, painless and which does not require contact with any part of the body^9^.

The treatment of chronic pain is complex and long, and leads to dependence on the health services and to high financial costs - apart from changes in the various social, physical and emotional aspects experienced during the same. Accordingly, there is a consensus that it is necessary to invest in integrative and complementary therapies which allow demedicalization and are low cost^10^. All the same, these therapies - described and recommended for use in clinical practice - must be tested through controlled clinical trials. 

Among the therapeutic resources currently described as integrative, complementary and holistic, used for the treatment and control of chronic pain, ear acupuncture (EA) has stood out. Based in the precepts of Traditional Chinese Medicine (TCM), this therapy uses a stimulation of auricular points, so as to harmonize the function of the organs and viscera of the human body^11^. EA has, therefore, preventive and curative aspects, as well as promoting relief of the signs and symptoms of different conditions. 

The scientific evidence centered on the effects of EA on chronic pain in the spinal column, however, is as yet limited due to the small number of studies involving this topic, as well as to the methodological shortcomings indicated in the same^12^. 

The objective of the present study was, therefore, to assess the action of EA on disability and tissue temperature in people with chronic pain in the spinal column.

## Method

This is a controlled randomized clinical trial^13^, of the parallel and blind type, undertaken between June 2015 and March 2016, in a university in Minas Gerais, Brazil. The population was made up of 535 people awaiting treatment in the institution’s physiotherapy clinic. 

The screening of the sample of volunteers with complaints of pain in the spinal column was undertaken by telephone. As a result, from the initial population, 149 people were excluded as they presented other types of pain. 

The following were established as inclusion criteria for selecting the sample: (1) age range between 18 and 80 years old; (2) presence of chronic pain in the spinal column for three months or over^14^, of any origin; (3) self-reported intensity of pain ≥ 4, on a numeric pain scale of 11 points^15^; and (4) availability to attend the EA sessions. The exclusion criteria were as follows: (1) individuals with infections, inflammation or injuries in the ear; 2) allergy to metal or to microporous tape; (3) undertaking previous energy therapy in the three months prior to the intervention; (4) receiving physiotherapy; (5) in continuous use of medication for pain relief; (6) refusal to receive the ear treatment through the use of needles and (7) pregnant women. 

**Figure 1:**
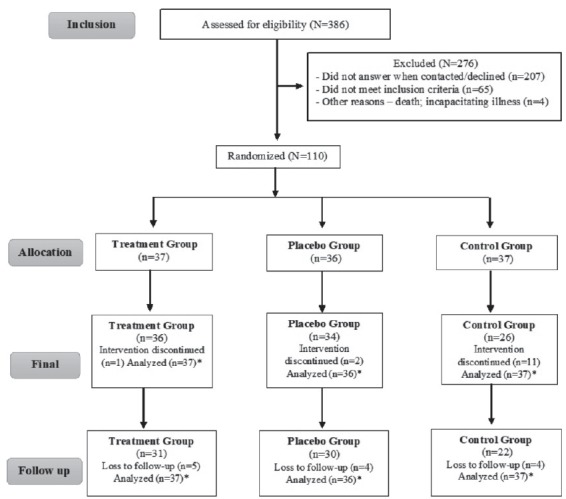
Flowchart of the participants involved in the study. Alfenas, MG, Brazil, 2016

The sample calculation was undertaken based on a pretest with 15 people (5 per group), using the GPower software, version 3.1, and BioEstat, version 5.0 software. A test power of 90% was adopted, as was a mean effect size (0.5) and a level of significance of 5%, resulting in the need for 30 individuals per group. So as to avoid sample losses, the calculation was corrected by 20%^16^. The eligible population obtained was of 110 individuals, and 83 concluded the study (Figure 1), with a loss of 27 (24.54%) individuals. 

*Analyzed through Intention-to-Treat.

The volunteers were randomly placed in three study arms: Treatment Group (n=37), Placebo Group (n=36) and Control Group (n=37). The randomization was made in four blocks, with approximately 27 people per block, by a researcher from outside the study, using the R software (version 3.1.1). Each number in a random sequence generated was placed in an opaque envelope and handed to the interventionist in the first intervention session. 

The assessments were undertaken at three points by the same trained investigators: before the first EA session, one week after the fifth session, and during the 15 day follow-up period.

The sociodemographic and clinical profile was determined by the following variables: age, sex, duration and cause of pain. The Rolland Morris Disability Questionnaire (RMDQ) was used to assess the interference of the pain in the activities of daily living^17^.

The RMDQ has 24 items with a total score varying from zero (no disability) to 24 (severe disability) and measures items related to the impact of the pain on the physiological functions (such as walking and movement, sleep and rest, appetite), psychological functions (irritation and bad mood) and social functions (interaction with other people, domestic and work activities)^5^. This instrument has been translated, adapted and validated to produce a Brazilian version^17^ and has appropriate psychometric properties^6^. 

In order to assess the tissue temperature of the cervical, thoracic and lumbar regions, images were taken using a thermographic camera (E-60 bx, ESTONIA) with resolution of 320 x 240 (76,800 pixels), in the spectral range of long-wave infrared radiation (7-13 μm) for dynamic study (60 Hz), with lenses with resolution of 25º x 19º, positioned horizontally at a distance of 3 m from the patient and 1 m vertically from the ground. For this assessment, the room was previously air-conditioned to 20°C for 20 minutes^18^. 

In order to take the thermographic photograph^18^, the volunteers remained at rest for 15 minutes and for the capturing of the image, remained in an orthostatic position, with the region of their backs uncovered and with the arms crossed in front of the chest. Double-sided polystyrene markers were attached against the following previously standardized points to assist in the assessment of the images^19^: cervical region (mastoid process, bilaterally; at the level of C7, bilaterally); thoracic region (acromion, bilaterally; free extremity of the 12^th^ rib, bilaterally); and lumbar region (free extremity of the 12^th^ rib, bilaterally; iliac spine, bilaterally). The thermographic images were analyzed using the FLIR Tools software, version 5.2.15161.1001.

In order to find the best therapeutic scheme for treatment, a protocol was created for intervention using EA, based in the recommendations of the Standards for Reporting Interventions in Clinical Trials of Acupuncture^20^, through which the following were defined: the duration of treatment, the number of sessions, and the devices and application points. This was subjected to an assessment process by five acupuncturists with over 10 years of experience in the area, and was then tested in a pretest. 

The EA treatment, in the Treatment and Placebo groups, was undertaken using semipermanent auricular needles, sterilized and disposable, 0.20 x 1.5 millimeters in size, of the Complementar Agulhas® brand. The ear was thoroughly cleaned beforehand using cotton and ethyl alcohol 70%. Next, the location of the auricular acupuncture points was confirmed using the *Acu-Treat* localizing device (DongBang®); and the needles were inserted and fixed with beige anti-allergic micropore. 

The auricular points for the Treatment Group were distributed according to the energy balance and according to the standards of TCM, and were applied in the following order: *Shenmen* (TF4); Kidney (CO10); Sympathetic nerve (AH6a); points for reestablishing energy balance, corresponding to one organ and one viscera; Cervical Vertebrae (HA13), Thoracic Vertebrae (AH11) and/or Lumbar Vertebrae (AH9), depending on the location of the pain^21^. 

In the placebo Group, a single point was applied, termed “Eye” (LO5). This point, sited in the center of the earlobe^21^, is far from the points applied in the Treatment group, and is not related to the focus of observation. 

Both groups received five sessions of EA, once a week, over a period of one and a half months, alternating the ear in each session. This time was defined based in the experience of the acupuncturists who assessed the treatment scheme proposed, as well as in the results obtained in the pretest. The entire procedure was undertaken by a professional who is specialized in acupuncture, with experience in the area for over three years^20^. 

The individuals who were allocated to the Control group, during the assessment period, received no guidance and no intervention. 

The blinding was applied to the study investigators and to the person who analyzed the results^22^; these people did not know to which group each volunteer had been allocated. 

The data collected were tabulated using Microsoft Office Excel*®* 2013, by two independent researchers, and their consistency was tested. For the statistical analysis, the Statistical Package for the Social Sciences, version 23.0, and BioEstat, version 5.0, were used. 

The data were analyzed through Intention-to-Treat (ITT), through the repetition of the values of the last assessment. For the sociodemographic and clinical variables, the Chi-squared tests and Kruskal Wallis tests were used. For the interclass assessment, we used the Kruskal Wallis test, followed by the Student-Newman Keuls test when necessary. For the interclass assessment, we used the paired Wilcoxon test. The level of significance adopted was of 5%. 

This study was approved by the Research Ethics Committee (CAAE N. 43818115.6.0000.5142/Opinion N. 1.041.266). Before the study began, the participants were assured of their right to receive the treatment at the end of the follow-up period, so as to comply with the ethical precepts and improve adherence. Thus, at the end of the study, the volunteers from the Placebo and Control groups received the same sessions of EA as the Treatment group.

## Results

A total of 110 individuals with chronic pain in the spinal column participated in the study. The comparison between the groups, according to the sociodemographic and clinical variables, indicates homogeneity between them - and, consequently, the adequacy of the randomization process, as shown in Table 1. 

**Table 1:** Characterization of the sample by age, sex, duration and causes of the pain. Alfenas, MG, Brazil, 2016 (n=110)

Variables		Groups			P Value
		Treatment (n=37)	Placebo (n=36)	Control (n=37)	
Age (x*±sd^†^)	(years)	47.5±13.9	51.0±14.9	46.2±15.7	0.272^‡^
Sex (%^§^)	Male	18.9	25.0	18.9	0.763^||^
	Female	81.1	75.0	81.1	
Duration of pain (x*±sd^†^)	(months)	47.5±13.9	51.0±14.9	46.2±15.7	0.850^‡^
Causes of the pain (%^§^)	Postural changes	16.2	44.4	16.2	0.057^||^
	Osteoarthritis	35.1	41.6	16.2	

*Mean; † Standard deviation; ‡Kruskal-Wallis test; §Percentage; ||Chi-squared test.

On assessing the impact of the auricular intervention on the disability resulting from the pain, through the use of the RMDQ, it was ascertained that statistically significant reductions were obtained for the Treatment and Placebo groups, between the initial and final assessments, and the initial and follow-up assessments. Furthermore, in the interclass analysis, and the final assessment, the Treatment group differed from the others, with a lower level of disability, as shown in Table 2. 

**Table 2:** Intraclass and interclass analyses of disability, expressed in median, (mean ± standard deviation) and confidence interval at 95%, in the three groups and at three points. Alfenas, MG, Brazil, 2016 (n=110)

Groups	Assessments		
	Initial	Final	Follow up
Treatment (n=37)	12.0*^†^ (11.8±5.8) 9.8-13.7	4.0*^‡^ (6.6±6.3) 4.4-8.8	6.0^†^ (7.5±6.7) 5.3-9.7
Placebo (n=36)	12.5*^†^ (12.8±7.0) 10.4-15.2	8.0* (9.9±7.2) 7.5-12.4	9.5^†^ (10.1±7.7) 7.4-12.7
Control (n=37)	11.0 (10.2±5.5) 8.4-12.1	11.0 (11.1±6.2) 9.0-13.1	11.0 (10.2±6.9) 8.6-13.2

*p < 0.05 between initial and final assessments (Wilcoxon); †p < 0.05 between initial and follow-up assessments (Wilcoxon); ‡p < 0.05 interclass analysis (Kruskal-Wallis, followed by Student-Newman Keuls).

There was a statistically significant increase in mean tissue temperature of the dorsal region, obtained through the infrared thermography, in the intraclass analysis (initial and follow-up/final and follow-up), as shown in Table 3. In the interclass analysis, the groups did not differ. 

**Table 3:** Intraclass and interclass analyses of the mean tissue temperature of the dorsal region, expressed in median, (mean ± standard deviation) and confidence interval at 95%, in the three groups and at three points. Alfenas, MG, Brazil, 2016. (n=110)

Groups	Assessments		
	Initial	Final	Follow up
Treatment (n=37)	30.4* (30.5±0.7) 30.2-30.7	30.2† (30.1±1.3) 29.6-30.5	30.9*† (30.8±0.3) 30.5-31.1
Placebo (n=36)	30.4 (30.4±0.9) 30.1-30.7	30.2† (30.1±1.2) 29.7-30.5	30.8† (30.6±0.8) 30.3-30.9
Control (n=37)	30.3* (30.1±1.0) 29.8-30.5	30.3 (30.2±1.3) 29.7-30.6	30.7* (30.5±1.3) 30.1-30.9

*p < 0.05 between initial and follow-up assessments (Wilcoxon); †p < 0.05 between final and follow-up assessments (Wilcoxon).

## Discussion

Chronic pain in the spinal column is incapacitating, principally when in the lumbar region^23^. Studies^19^ ^,^ ^24^ have demonstrated that the level of disability varies proportionally to the intensity and to the pain threshold, and causes limitations in daily activities (such as difficulties in dressing, sitting, standing up, walking and lifting objects), changes in sleep and constant worry, as well as absenteeism from work^25^. It is, therefore, necessary to identify interventions capable of reducing the disability of people with chronic pain in the spinal column. In this context, the present study corroborates that ear acupuncture is efficient in minimizing this disability. 

One clinical improvement in disability caused by pain processes is obtained if the RMDQ score reduces from the baseline by 30% and the pain in the spinal column is classified as better on a global evaluation scale^26^. This study’s volunteers who received the EA protocol for chronic pain in the spinal column experienced a reduction of 66.66% in the level of disability in the final assessment, compared with the initial assessment. This effect lasted 15 days, until the point of the follow-up assessment, at which a reduction of 50% in the levels of disabilities was observed. 

Equally, other researchers^27^ ^-^ ^28^ have observed improvement in functional lumbar capacity with treatment undertaken using EA. For Traditional Chinese Medicine, lumbar pain is a syndromic manifestation related to energy changes of organs such as the kidneys. As a result, the stimulation of this and of other auricular points indicated as calming and analgesic, such as that undertaken here, is reflected through the whole organism, helping to reestablish the energy balance of the body as a whole^29^.

In the placebo group, the reduction in disability was below that of the volunteers who received the treatment, demonstrating that true EA is significantly more efficacious than the simulated treatment, which corroborates the results of studies^27^ ^-^ ^28^ that have also shown that the score for disability in the RMDQ reduced most in the group that received the true intervention, when compared to the placebo group.

The effects in the placebo group cannot, however, be ignored. These effects can be explained due to the possible psychological effects which may appear due to the volunteers’ expectations in relation to the therapy, which is termed the “placebo effect”^30^. Indeed, studies have indicated that pain influences many dimensions apart from the physical aspects, such as that the psychological factors^31^ ^-^ ^32^.

The protocol used in this study promoted the relief of the chronic pain in the spinal column and, consequently, an improved microcirculatory profile in the area affected, which may be highly desirable for reestablishing the chronic pain conditions. Some authors^33^, who have already analyzed the patterns of skin temperature in musculoskeletal disorders, have also found an increase of this in the place affected by the pain, during the treatment of temporomandibular disorder. 

The changes of temperature in specific regions of the body can indicate physiological changes^34^ ^)^ and may be used as a parameter in assessing painful conditions. Therefore, infrared thermography is an appropriate method for studies investigating such phenomena, as it provides images of distribution of the temperature of the skin of the body, which is conditioned by the activities of the microcirculation^35^. 

In cases of chronic injuries, such as - for example - in the myofascial dysfunctions, hypo-radiant areas can be detected in infrared images. This happens because these conditions can cause greater muscular activity at rest, which results in an increase in the intramuscular pressure and, consequently, greater mechanical compression of the blood vessels of the muscles in the region affected; over time, the reduced blood supply leads to a reduction in skin temperature^36^ ^-^ ^37^. 

Nevertheless, specifically in the case of chronic pain in the spinal column, no evidence available was found in the literature reporting possible clinical effects of EA on skin temperature, meaning that the present results were pioneering in the investigation of this phenomenon. 

EA, therefore, is a therapeutic resource that is easy to apply, low in cost and with a relative absence of side effects, which assists in reestablishing health as a whole^29^. In addition to this, it may be applied, multi-professionally, at all levels of healthcare. In this scenario, upon evidencing the high prevalence of people with chronic back pain^1^ ^)^ and the negative impacts that this causes on their lives^2^ ^-^ ^4^, EA may be seen as an intervention tool which may be easily implemented in the nurse’s clinical practice, so as to help these people’s rehabilitation and, consequently, improve the quality of their lives. 

As possible limitations of this study, one may consider the withdrawal of some volunteers from the treatment and placebo groups due to the discomfort caused by the insertion and placement of the semipermanent needles in the ear. It is believed, furthermore, that the fact that the volunteers from the control group remained for a considerable period of time receiving only the assessments may have caused a greater loss in this group, although they were assured that they had the right to receive the intervention at the end of the follow-up period. Furthermore, the intervention was undertaken individually, as is strongly recommended by TCM, and this hinders the use of the same auricular points, although satisfactory and statistically significant results have been obtained in this way.

Further studies are suggested, in different contexts, to improve the substantiation of these results, as well as investigating the action of EA on other behavioral and physiological variables of chronic pain related to the musculoskeletal system. 

## Conclusion

There was a statistically significant difference between the groups and over time, in relation to physical disability; and in relation to tissue temperature, this difference occurred during the follow-up time. These findings demonstrate that EA is an intervention that may be implemented in the nurse’s clinical practice, so as to assist the treatment and, consequently, the rehabilitation of people with chronic pain in the spinal column. It is hoped that the results of this study will encourage the use of this therapeutic resource by nurses in their care activities. 
